# Perception and belief in oral health among Karen ethnic group living along Thai-Myanmar border, Thailand

**DOI:** 10.1186/s12903-020-01318-w

**Published:** 2020-11-11

**Authors:** Sai Wai Yan Myint Thu, Yaowaluk Ngeonwiwatkul, Pannamas Maneekan, Suparat Phuanukoonnon

**Affiliations:** 1 Department of Social and Environmental Medicine, Faculty of Tropical Medicine, Mahidol University, Bangkok, Thailand; 2 Department of Community Dentistry, Faculty of Dentistry, Mahidol University, Bangkok, Thailand; 3 Department of Tropical Hygiene, Faculty of Tropical Medicine, Mahidol University, Bangkok, Thailand

**Keywords:** Oral health, Qualitative research, Border population, Thailand

## Abstract

**Background:**

Utilization of oral health services has been low among rural populations in Thailand, especially for minority ethnicity populations living along the borders. The aim of this study was to increase understanding of the determinants of the underutilization of oral health services among these populations.

**Methods:**

A qualitative study using in-depth interview and semi-structured interview was conducted among participants of Karen ethnicity living in Mae Tan Sub-district, Thasongyang District, Tak Province, Thailand. The interviews focused on exploring the participants’ perceptions of oral health problems, oral health and hygiene, and oral health-seeking behaviors. The verbatim-transcribed interviews were analyzed using thematic analysis.

**Results:**

A total of 101 participants (50 adults and 51 children) with a Karen ethnic background took part in the interviews. Most participants could not identify oral health problems and did not perceive dental disease as a problem unless there was severe pain that could not be relieved by painkillers. The Karen ethnic community worked as subsistence farmers, and their busy daily activities consisted of farming, going to the forest to hunt and gather, performing housework, and taking care of their children. Dental health was given a lower priority compared with general health. The perceived value of primary teeth was low, which was identified as an underlying factor resulting in delayed oral health care seeking among this population. The participants had relied on self-care throughout their lifetimes, using either traditional medicines or modern painkillers to relieve toothaches. Fear of dental procedures among children was also described as a barrier to seeking dental health care.

**Conclusions:**

This study found that the lifestyle and traditions of Karen people living in this area influence their oral health care and hygiene activities as well as their health-seeking behaviors. Further research should emphasize how to improve oral health promotion by providing necessary services and health education appropriately to Karen ethnic populations living along the Thai-Myanmar border.

## Background

In Thailand, there are a number of barriers to accessing oral health care among people living in remote areas [1]. Some oral health services are not covered by the Thai universal health insurance; this can discourage these people’s oral health service utilization because they may not be able to afford a visit to the government hospital when they have oral health problems [2]. Despite being beneficiaries of the Thai universal health insurance, most people of Karen ethnicity in Thasongyang District seek professional oral health care mostly when oral health problems become severe as chronic periodontitis, acute pulpitis, pulp necrosis, and periapical abscess were included in the most common causes of the dental clinic visit at Thasongyang hospital between 2016 to 2019[3]. Delayed oral health seeking can result in many negative consequences, including pain, abscess, and cellulitis, which lead to the need for high-cost specialized treatment, causing financial burden for low-income communities [4].

Previous qualitative studies have explored knowledge, perceptions, beliefs, and oral health care seeking among minority communities [5–10]. However, studies regarding the folk knowledge, perceptions, and oral health-seeking behaviors of populations living along the border areas of Thailand are lacking, despite high rates of oral health problems among these people. Exploring the reasons for low oral health utilization in these communities require an understanding of community awareness, knowledge, perceptions, and beliefs regarding teeth, dental illness, and oral hygiene, which can influence the oral health care-seeking behaviors among this population in Thailand. Furthermore, such an understanding will provide useful information for future oral health promotion programs in these communities. The present study explored folk oral health knowledge, perceptions, and oral health care-seeking behaviors among the population of Mae Tan Sub-district, Thasongyang District, Thailand.

## Methods

### Study design and study population

Qualitative research approaches using in-depth interviews and semi-structured interviews were employed to explore and understand the various personal and social environmental factors influencing participants’ beliefs, perceptions regarding oral health, oral health-seeking behaviors. The study was part of the pre-assessment component of the Health Promoting School intervention project entitled the “Buddy Brushing program.” This program was implemented in five schools (Mae Tan School, Mae Po School, Khun Houy School, Uhu School, and Tung Tam School) in the selected villages (Thai Clinical Trials Registry No: TCTR20190221002).

**Fig. 1:**
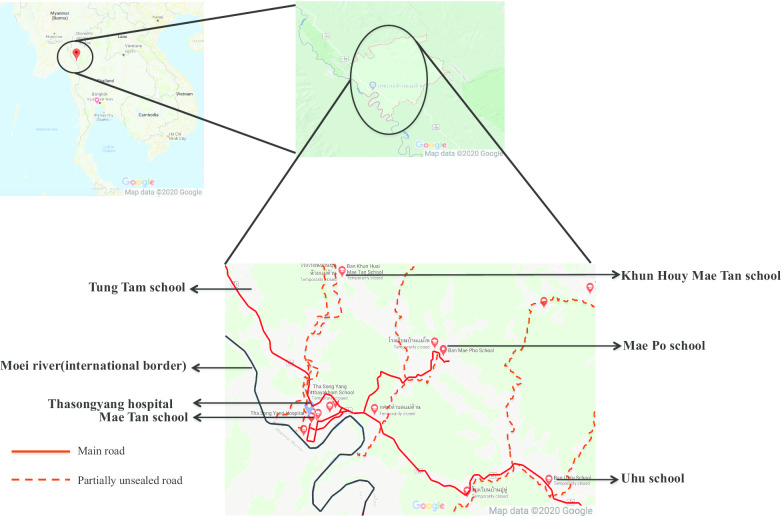
Map of Mae Tan Sub-district, Thasongyang District, Tak province. Map was modified from Google maps [11]

Interviews were conducted in these five selected villages (of 10 total villages) in Mae Tan Sub-district that were in Thasongyang Hospital’s catchment area for the provision of routine oral health services and oral health promotion activities in schools and communities. Of the five selected villages, two were remote-rural villages (Khun Houy Mae Tan and Mae Po villages), two were rural villages (Uhu and Tung Tam villages), and one was town village (Mae Tan Village) (Fig. 1). From each village, 10 parents/guardians who were responsible for children’s oral hygiene were selected by purposively sampling using three age strata (< 30 years: 3 participants, 30–45 years: 4 participants, > 45 years: 3 participants), and 10 children aged 9–13 years from each village were also invited to participate in the study. The in-depth interviews were conducted with adult participants and semi-structured interviews were conducted with children. Village health volunteers served as gatekeepers, identifying the first few potential participants. The snowball sampling technique was then used to recruit the rest of the participants. All participants provided written consent or assent before participating in the interviews.

### Data collection

The research team developed the interview guide for both in depth interview and semi-structured interview session (See Additional file 1). The interview guide for in-depth interview consisted of questions on the participants’ current oral health conditions, perceptions regarding oral health problems, beliefs about the causes of oral health problems, norms and traditions for oral hygiene practices and oral health-seeking behaviour, and reasons for delaying or avoiding seeking professional oral care. For semi-structured interview, the content in the interview guide was similar to the in-depth interview guide, however; the semi-structured interview approach was different from those of in-depth interview. The interviewer (SWYMT) was a male doctoral student in social medicine track of Tropical Medicine program and had an interest in dental public health and health behaviour change. The interviewer had an educational background in dentistry (B.D.S.) and public health (M.Sc.) and received training in conducting in-depth interview, semi-structured interview and qualitative data analysis by an experienced qualitative researcher (SP) before visiting to the field. There was no existing relationship between the interviewer and the participants before conducting the study. The research team acknowledged the difficulty of engaging children in the interview process and noticed that the question–answer format can be less successful than entering into extended conversation [12]. The research team therefore adopted a visual/arts-based narrative method using photos (such as dental caries conditions, toothpaste brands and sweet food and drinks) as this method was considered more child friendly and would encourage children to express themselves more openly [12]. A digital voice recorder was used to audio-record the interviews, and field notes were also taken during the interviews.

After conducting a pilot study with five villagers in Mae Tan Sub-district, the interviewer and the village health volunteer (Mr. Wanchai Kulabkeeree), who served as an interpreter, visited the participants’ households to conduct in-depth interviews with the adults and went to schools to conduct semi-structured interviews with the children. For adult participants, the researcher conducted interviews when participants were free and convenient during the day; and for child participants, the interviews were conducted after school. The interviewer explained about the aim and rationale of the study to both adult and child potential participants and invited them to participate in the study. Before starting the interview, demographic information of the participants was obtained through questionnaire developed by the research team (See Additional file 2). Because all participants were of Karen ethnicity, the interpreter interpreted between Karen or Thai and Burmese (the interviewer’s first language) during the interview sessions. The individual interviews lasted a maximum of 40 min.

### Data analysis

The Burmese audio files were fully transcribed into English. The interview transcripts were then transferred to the RQDA package of R statistical software [13] for thematic analysis. Transcriptions uploaded onto RQDA were analyzed progressively to recognize when data saturation was reached. Initial coding of the transcripts was performed while reading the transcripts line by line (by SWYMT). Initial codes were then modified by combination or deletion. Themes were developed from the codes on the basis of the research objectives of the study. Finally, the qualitative research report was prepared through the integration of the findings from the in-depth interviews. Information recorded in the field notes was used to triangulate the findings of the in-depth interviews. Main findings are summarized in Table 1 below.

**Table 1:** Perception and belief of oral health among Karen ethnic group

Themes	Main findings
Folk knowledge of teeth	Two sets of teeth: low perceived value of baby teeth compared with adult teeth. Baby teeth are susceptible to tooth decay regardless of oral hygiene practices and regular dental visit
Folk knowledge and perceptions about Dental caries	Identification of dental caries was based on pain rather than on external appearance such as black staining Tooth worms as a main cause of dental caries Sweet foods and drinks strengthen the worms, which creates holes/cavities in tooth
Folk knowledge and perceptions about Gingivitis	Identified as gum bleeding Caused by brushing teeth too fast, for a long time, or too hard, or irritation from chewing betel quid Inadequate knowledge about cause of gingivitis Not consider as a problem unless they see large amount of bleeding
Sweet food consumption as a risk factor for dental problems	Knowledgeable about relationship between consumption of sweet foods or drinks and dental caries Recent increase of sweet foods or drinks availability as the main cause of high prevalence of dental caries among children
Oral hygiene practices	Gargling with drinking water and using toothpicks after meals to remove leftover foods were common practices Toothbrush and salt were commonly used for brushing teeth Toothbrushing while taking a bath in the evening was sufficient, regardless of having dinner after toothbrushing Purpose of toothbrushing was for cleanliness and did not worry about health risk of not brushing their teeth twice a day and before going to bed Inadequate knowledge of mouthwash and dental floss, only available in town areas, therefore not accessible by most participants
Poor awareness regarding seeking dental care	Poor awareness of preventive dental care services at the dental clinic of Thasongyang hospital Consider seeking dental care as lower priority than seeking general health care due to ability to deal with dental problems by themselves Reliance on self-care for oral health problems from the annual visit of mobile dental clinics to their villages
Ability to self-treat dental illness	Lack of or insufficient oral hygiene tools and oral health care facility in the past lead to reliance on self-care for their oral health problems For dental pain, wait to resolve dental pain on its own or brush more frequently and limit consumption of sweet foods or take analgesics or take traditional remedies. If pain was not resolved, they visited to dental clinic For gum bleeding, rinse their mouths with normal water, warm water, or salt water For tooth mobility, avoid discomfort or pain while eating by chewing food on the other side of the mouth or eating soft diet Decision to seek professional dental care was based on the poor outcome of the self-care rather than on their awareness of the need for dental treatment
Seeking professional dental care	Due to suffering severe dental pain that interfere with work-related activities or their daily life For children with dental pain, parents let them to wait until the hospital team to visit their school or wait for mobile dental clinics or parents sent their children to school hoped that the teachers would take the children to the hospital Parents have little extra time to accompany with their children due to busy lifestyle of parents or guardians
Fear of dental care procedures	Fear of dental procedures and equipment and fear or tooth removal lead to self-care of oral health problems Unpleasant experiences of dental visit cause vicious circle of lack of timely care-seeking behavior and dental fear

## Results

### General characteristics of the participants

In total, 101 people (51 children and 50 parents or guardians) participated in the semi-structured interviews and in-depth interviews. There were no participants who refused to participate or withdrew consent. The adult participants’ ages ranged from 24 to 63 years, and most adult participants were women (94%). The majority of participants were Buddhist (96%), and all belonged to the Karen ethnic group. In terms of education, 40% of the participants had finished secondary school or higher, 33% had finished primary school only, and 24% reported having no education. Most of the parents were farmers (61%), and 22% were housewives, traders, or casual laborers.

The age of the student participants ranged from 9 to 13 years, and they were in the fourth or fifth grade. Equal numbers of boys and girls were selected for participation in the study. Regarding the students’ schools, 11 of the students participating in the in-depth interviews attended Tung Tam School, and the remaining 10 students were from the other four schools.

### Folk knowledge of teeth

In terms of oral health knowledge, most of the participants knew about two sets of teeth: baby teeth (*Fun Nam Nom* in Thai or *Mae Nu Tee* in Karen, both meaning “breast milk teeth”) and adult teeth or permanent teeth (*Fun Tae* in Thai or *Mae Noh Kor Jar* in Karen, both meaning “true teeth”). The majority of the participants perceived baby teeth to have low value because of their short-term utility and because they were only for chewing foods. They thought that permanent teeth were stronger and more effective in chewing foods, as adults ate a greater variety of foods, especially foods that were tougher to chew. Moreover, permanent teeth were seen as irreplaceable and needing to last many decades.

After losing baby teeth, we simply throw them away. But adult teeth, we cannot lose them, as they are more important and necessary. We use them throughout our lives. (30–45-year-old woman, Mae Tan Village)

Therefore, the participants reported paying less attention to protecting baby teeth, which contributed to the development of tooth decay and toothache. In fact, most participants had experienced dental caries in their baby teeth. They thought that baby teeth were weak and prone to tooth decay, which they saw as occurring spontaneously, regardless of oral hygiene or regular dental clinic visits.

### Folk knowledge and perceptions about dental illnesses

Traditionally, Karen people believed that most general illnesses occur as a result of an attack by an offended spirit, the loss of the soul, or sorcery. However, among the participants in this study, dental problems were not generally perceived as having supernatural causes. The participants identified dental problems symptomatically, for example as toothaches, bleeding gums, sensitive teeth, or tooth mobility. Only dental caries was regarded as a disease. In the study villages, the most common dental health problems were dental caries and toothache.

#### Dental caries

Dental caries (*Mae Ka Or* in Karen) were described both in scientific terms (“tooth decay”) and using Thai phrases translating as, for example, “blackened teeth” or “dental hole/cavity” or Thai and Karen phrases meaning “tooth worms” or “worms eating the tooth.” The participants’ understanding of the etiology of dental caries came from a combination of local/traditional beliefs and scientific knowledge. However, most adult and child participants mentioned “tooth worms” as a local name for dental caries (*Mang Kin Fun* in Thai or *Mae Ka Or* in Karen), and these worms—tiny biological organisms that ate the tooth material—were seen as a main cause of dental caries. The participants thought dental caries resulted from being infected by this worm.

Consuming a large quantity of sweet foods or drinks and having food stick to the tooth surface were believed to be predisposing factors creating the worms. Because these worms liked to eat sweet food, consuming a large amount of sweet foods was seen as strengthening existing worms on the tooth surface. Holes/cavities in teeth were understood as resulting from the teeth being damaged by these worms.

Identification of dental caries was described as being based on people’s external appearance, such as having “black teeth”; however, this might not actually be applicable for this study population. Among adults, dental caries mostly occurs in the posterior teeth, where it is difficult to see without checking carefully in the mirror. In addition, most of the adult participants chewed betel quid, making it impossible to notice additional black staining on the tooth surface because of betel quid staining. Most participants mentioned that they did not actively check their teeth. They initially recognized oral diseases after experiencing an uncomfortable feeling, pain, or obvious swelling of the face.

I visited the dental clinic because I felt that something was stuck inside my tooth, very uncomfortable. It had been for some time, and I realized that I may have “Mang” eating up my tooth and there is cavity or hole in my tooth. (30–45-year-old woman, Mae Tan Village)

#### Gingivitis

Of the participants who experienced bleeding gums, most noticed this during toothbrushing. They thought that bleeding gums resulted from brushing their teeth fast, for a long time, or too hard. None of the participants knew that inadequate oral hygiene and the presence of dental plaque between the teeth were the true causes of bleeding gums. Most of the interviewed children had heard about plaque in health education classes, but they were unable to relate plaque to gingivitis. Gum bleeding was generally regarded as a symptom rather than a disease, with some participants viewing bleeding gums as consequences of prolonged toothache or severe dental caries.

Some adult participants thought that irritation from chewing betel quid, which was a common practice among the adult population, was one of the causes of bleeding gums. None of the participants thought that gum bleeding was a problem, although some worried if they saw a large amount of bleeding during toothbrushing.

Yes, I think it is normal about toothbrushing and bleeding gums. I notice bleeding when I rinse my mouth. But it only bleeds very little—not a problem. It is irritated from brushing too hard, I guess. (30–45-year-old woman, Mae Tan Village)

#### Sweet food consumption as a risk factor for dental problems

Common sweet foods, snacks, and drinks in the study villages were chocolate, candy, cakes, cookies, ice creams, artificial fruit juice, and soft drinks (e.g., Fanta, Coca-Cola, and Pepsi). Most consumers of these products were children, who bought sweet foods and drinks from the local shops at costs ranging from US$ 0.03 (for a piece of candy) to US$ 0.3 (for a piece of cake). The most popular items were ice creams, Deedo sweet drinks (250 cc), and soft drinks (250 cc), each of which cost US$ 0.2.

Most participants knew about the association of the consumption of sweet foods and drinks with oral health problems. Some said that, according to health education classes, the consumption of sweet foods or drinks would not cause any problems if they brushed their teeth after consuming these products. Few of the student participants mentioned that milk and natural foods such as fruits and vegetables were good for dental health. Fruits and vegetables were easily accessed and mostly free of charge because the local people grew fruits and vegetables in their gardens for their own consumption.

The teacher taught me how to brush my teeth after eating meals or snacks, but they’ve never taught [us] exactly about what food is good for the teeth. (9–13-year-old boy, Khun Houy Village)

If we eat a lot of fruits, our teeth will be cleaned, and we will not have tooth decay. (9–13-year-old girl, Uhu Village)

According to the adult participants, when they were children, they were only able to access natural foods such as fruits and vegetables because sweet foods and drinks were not available in their villages at that time. However, sweet foods and drinks were widely available at the time of the research, and it was believed to be a main cause of the high prevalence of tooth decay among the children in this area.

I had three siblings when I was young, and nobody had tooth decay. We did not eat sweet foods. Now, there are a lot of sweet foods and drinks available in this village. Children like those sweets. That is why most of the children have tooth decay. (30–45-year-old woman, Mae Tan Village)

#### Oral hygiene practices

Most participants used their own oral hygiene methods, such as gargling with drinking water and swallowing after meals. Gargling was a common means of removing food remaining in the mouth or stuck between the teeth. However, this gargling normally happened while drinking water after a meal. Gargling with water was practiced inconsistently: people did not gargle after every meal, and they gargled more or less depending on the amount of food left in their mouths. For example, they reported that swishing one or two times was sufficient to remove a few pieces of food. Rinsing the mouth with water after consuming meals or snacks was not properly taught in health education classes. Using toothpicks was very common among the adult participants when gargling failed to remove food stuck between their teeth.

Toothbrushing was among the general oral hygiene practices reported. Traditionally, Karen people used a finger to rub their teeth with salt as a means of cleaning their teeth. Although toothbrushes were easily accessible during the research period, toothbrushing using a toothbrush and salt remained very common because toothpaste was unaffordable for some households.

Brushing with salt is actually very good. It can increase the stability of the gums and teeth. It can also reduce bad smells in the mouth. (30–45-year-old woman, Khun Houy Village)

Now I brush my teeth with toothpaste. During my childhood, I didn’t have money to buy toothpaste, so I brushed my teeth with salt. (< 30-year-old woman, Uhu Village)

Most adult participants agreed that oral hygiene practices had changed since they were children. Toothbrushes were introduced in the study villages in the late 1980s, initially for primary school students. At the time of the research, children in the area learned how to brush their teeth for the first time in their first-year kindergarten class when they were 5 years old. Although some children might learn about toothbrushing earlier at home, most of the students participating in this study reported learning how to brush their teeth correctly at school. Toothbrushes were given to all students in schools annually, in line with a national policy, and toothpaste was available in schools for use after eating lunch.

All participants believed that toothbrushing once per day was sufficient. In the Karen villages in the study area, the bathroom was a separate building located approximately 5–20 m away from the main house. Personal hygiene tools such as soap, toothbrushes, and toothpaste were kept in the bathroom. Toothbrushing while taking a bath was a common practice, and the participants reported taking a bath once a day in the evening. The purpose of toothbrushing for the participants was mostly cleanliness and hygiene, and they did not worry about the health risks of not brushing their teeth frequently enough.

I take a shower once a day in the evening, and I also brush my teeth at the same time. It is convenient; everyone in my family does the same. Then I have dinner and go to bed. But to brush our teeth again after dinner, we would have to go to the bathroom to brush our teeth, and it is inconvenient. We rinse our mouths after dinner, and that is enough. (30–45-year-old woman, Khun Houy Village)

Most adult participants had not heard of mouthwash or dental floss. These oral hygiene products were covered in school, and they were commercially available in pharmacy shops and in a convenience store in town, but very few participants—mostly those living in the town—had used them.

With the recent introduction of dental floss in schools [14], the children were aware that toothbrushing and using dental floss resulted in more effective teeth cleaning compared with toothbrushing alone. Additionally, a few children used mouthwash, saying that using mouthwash after toothbrushing resulted in better oral hygiene.

#### Poor awareness regarding seeking dental care

Most adult participants thought that the condition of their teeth was good, even when they had a mild toothache or slight tooth mobility. Generally, they experienced suffering less frequently from dental diseases than from other health issues. Therefore, they considered seeking dental health care a lower priority, compared with general health care. Moreover, there were no oral health care facilities located near their villages, and most participants relied on their own methods to take care of their oral health problems between the annual visits of the mobile oral health clinic to their villages.

The participants knew that there was a dental clinic at the hospital and that its services were for treating dental illnesses, but they were not aware that the clinic also provided regular check-ups to prevent dental diseases. Some male participants visited the dental clinic for the first time when they had serious oral health problems, but most female participants first visited the dental clinic as part of a routine pregnancy check-up at the hospital. After their first oral health check-up during pregnancy, none of these women returned to the dental clinic for additional check-ups. They thought that this initial check-up during pregnancy was a health care requirement of pregnancy.

I visited the dental clinic as a part of a check-up when I was pregnant. Now, I don’t go to the dental clinic, because I am not pregnant. So it has been 4 years since I last visited the dental clinic. (30–45-year-old woman, Mae Tan Village)

Most children received their first oral health check-up when they enrolled in school. Oral check-ups were regularly conducted twice a year, at the beginning of each semester. The parents/caregivers participating in this study did not know that they should take their pre-school-aged children to the dental clinic for check-ups; therefore, they did not consider it a delay in care if their children received their first dental examination and services only after starting school.

The low priority these research participants placed on oral health care can be explained by their perceptions of their ability to deal with dental problems on their own and by their satisfaction with the existing mobile community oral health program implemented through Thasongyang Hospital.

#### Ability to self-treat dental illness

The adult participants mentioned that they had experienced a disadvantage during their childhood, which included a lack of professional dental care and oral hygiene tools, and they therefore had to rely on themselves to solve their oral health-related problems. Most of the dental pain they experienced was intermittent. Therefore, even when they suffered pain, some participants waited for the pain to resolve on its own because they believed that dental pain would disappear spontaneously without professional care. Most participants had confidence in treating these problems themselves by taking pain-relieving medications when they experienced toothache. If the pain was very severe and was not relieved by these drugs, they went to the dental clinic. Therefore, the decision to seek professional dental care was based on the outcome of the self-care rather than on their awareness of the need for dental treatment.

Pain comes and goes, and it is normally relieved even if I don’t do anything. I have only visited the dental clinic when I suffered from severe pain. If I have a bad toothache, I cannot eat and even cannot sleep, that is the time I need to see the dentist. (9–13-year-old boy, Mae Po Village)

Most of the time, toothache pain is mild, and I can endure it. If it is severe, I take paracetamol. I will wait for the dentist to come to my village to check. But if the pain is still severe, of course I will see the doctor at the hospital. My neighbor waited until he had an abscess in his gum—very bad, as we cannot treat abscess by ourselves. (> 45-year-old man, Tung Tam Village)

When suffering with severe toothache or swelling in the oral cavity, the participants applied traditional remedies to relieve the pain. To remove *mang*, the traditional healer used oil to rub the swollen cheek and recited a Karen mantra. Salt, charcoal, and ashes were used as traditional remedies to relieve pain and remove black stains from teeth by rubbing the teeth with a piece of charcoal, rinsing with saline water, and then rubbing the teeth with ashes. When the toothache or swelling was not reduced by traditional medicine, the participants tried modern medications, using multiple medications at the same time or one after another, based on the improvement in the pain or the accessibility of the drugs.

There are many herbs. My grandmother used some leaves, but I use red onion. When we have a toothache, we put red onion inside the dental holes/cavities. In this way, we can relieve the toothache. (< 30-year-old woman, Khun Houy Village)

*“He really can relieve pain. He has never visited the dental clinic. He just uses Burmese traditional medicine [Bain Daw Say].”* (30–45-year-old woman, Uhu Village).

Some participants believed that brushing their teeth more frequently and limiting their consumption of sweet drinks would relieve dental pain. Some reported rinsing their mouths with normal water, warm water, or salt water to stop gum bleeding. Tooth mobility was caused by long-term betel quid chewing. This problem was found among adult participants, who reported avoiding discomfort or pain while eating by chewing food on the other side of the mouth or eating a soft diet.

I have tooth decay, but it does not cause any pain. But mobility of the teeth is not good because my mother suffered pain while chewing food because of tooth mobility. She waited for some time and then decided to visit the dental clinic because she could not chew food properly anymore. (30–45-year-old woman, Tung Tam Village)

#### Seeking professional dental care

More than half of the adult participants had never visited a dental clinic. Those who had visited a dental clinic did so because their self-care efforts failed to relieve their pain and because their dental diseases impacted their work-related activities or interfered with everyday life. There were two approaches to caring for children with dental problems: Their parents either brought these children to the hospital, or they sent their children to school and hoped that the teachers would take them to the hospital. Most parents and guardians had busy lifestyles; they had many work commitments in the fields or farms that were necessary for earning income, as well as housework that included taking care of young children. They normally worked 7 days a week; therefore, they did not have extra time to accompany their children to the dental clinic. The parents and guardians participating in this study thought the school did a better job than they could in terms of taking care of their children’s health, including their oral health.

But I was very busy with my work. That is why I didn’t have time to bring my children to the dental clinic. They can get a check-up at school, and it is sufficiently good. If they are sick, I send them to school anyway; the teacher will take care of them and bring them to the hospital. (30–45-year-old woman, Khun Houy Village)

I have never sent my child to the dental clinic. The dentist and the team from the hospital regularly come to the school to examine my child for oral health problems and deliver dental health education to my child. They do a better job, and I am always very busy with work. (30–45-year-old man, Tung Tam Village)

Instead of visiting the dental clinic, children with mild toothache preferred to take pain-relieving medication and wait for the hospital team to visit their school, which they did regularly every 2–3 months. Similarly, adult participants also preferred to wait for the mobile oral health service program to come to their village.

I advise my child to wait for the doctor because I believe that the doctor will give better drugs when the hospital team comes to the school. Otherwise, the teacher will bring her to see the dentist at the hospital. (30–45-year-old woman, Uhu Village).

After they had received treatment at the dental clinic and their pain was relieved, the participants did not pay attention to taking care of their treated teeth. For example, if they lost a filling, they did not return to the dental clinic to replace it.

#### Fear of dental care procedures

All children received oral health examinations in school. Most children participating in this study expressed fear of dental procedures and equipment. They were afraid of experiencing pain during dental procedures, and they also feared that their teeth would be removed. Some children had heard negative stories about their friends’ dental visits, and others had their own unpleasant experiences. Their experiences with local anesthesia injections using needles and with the removal of teeth using dental forceps resulted in a vicious circle of lack of timely care-seeking behavior and dental fear. Some participants pointed out that one reason for attempting self-care for dental problems was to avoid the dental clinic because of fear.

## Discussion

The majority of the people living in Mae Tan Sub-district are of Karen ethnicity and have their own traditions regarding responding to illness. In the first stage of the traditional method of dealing with dental illness, family members try to identify the symptoms and attempt to reduce them with traditional medicine, or they may ask someone who has had a similar illness for assistance. If they cannot relieve the symptoms, they may attempt to use several traditional or modern treatments together or one after another, depending on the progress of the patient’s symptoms, availability of the treatment methods, and cost [15].

In line with prior reports on caregivers from minority groups in different countries [6, 8, 10, 16], most participants in the present study placed low value on primary teeth. This might be because of poor knowledge regarding the importance of primary teeth among parents and caregivers in the study area. When the parent/guardian participants identified a particular oral health problem, they preferred to wait for the primary teeth to fall out by themselves rather than visiting the dental clinic; this finding was also reported in a previous qualitative study among low-income caregivers [9].

Poor knowledge regarding dental caries and gingivitis among the participants in this study was indicated by their failure to notice oral health problems until they experienced discomfort or pain. Additionally, they were unable to examine their mouths for abnormalities. These findings were consistent with a similar study among the primary caregivers of low-income Latino pre-school children, which concluded that these caregivers lacked awareness of their children’s dental health problems until the children complained of continuous pain [17]. Additionally, another qualitative study in the small rural U.S. city among Mexican migrant caregivers reported that both the visual identification of black staining on teeth and complaints of pain were required for caregivers to decide to take their children to a dental clinic [10]. Poor awareness regarding dental health problems might result from a lack of regular oral health check-ups at a dental clinic or from insufficient knowledge to identify dental diseases. Although the participants in the present study had poor knowledge regarding the causes, signs, and symptoms of dental diseases, they had adequate knowledge about which foods and drinks are bad for dental health. This finding was similar to the results of a previous study among mothers of pre-school children in Bangkok [18].

Regarding oral hygiene practices, gargling with water and using toothpicks were common methods for removing food debris in the oral cavity because mouthwash and dental floss were not easily accessible or affordable for most participants. This finding was consistent with a study conducted among the Burmese community in the U.S., many of whom reported never having seen used or even seen dental floss or mouthwash [19]. The participants in our study thought that toothbrushing could help with teeth cleaning, but they were not aware of the health benefits of toothbrushing. Although children learned about toothbrushing in school, they still believed that brushing once a day was sufficient. This could be explained by the local practice of toothbrushing during bathing, which they learned from their parents. This finding indicates the importance of oral health education for parents/caregivers to facilitate taking proper care of their children’s oral health.

The Karen ethnic community’s low utilization of professional dental care available in the hospital may be explained by their confidence in their ability to self-treat dental illnesses. This research also identified the lack of a tradition of visiting the dental clinic. Many studies showed that the parents/guardians had experienced hardships in their lives, including having insufficient money to buy oral hygiene tools and a lack of access to oral health services; therefore, they had to use home remedies or traditional medicines rather than visiting the dental clinic [7, 9, 15, 20]. Tooth mobility was a common oral health problem among the adult participants in this study, possibly because of clinical attachment loss caused by long-term betel quid chewing [21]. Scientific evidence has shown that betel quid contains areca nut extracts (predominantly arecoline) and slaked lime, which can damage oral tissues by impairing the function of periodontal fibroblasts and increasing the production of reactive oxygen species [22, 23]. Although their teeth were mobile and painful while chewing food, the participants tried to relieve discomfort by chewing on the other side of the mouth or eating soft foods. In addition, parents/caregivers in this study were not aware of the recommended timing for the first dental visit for their children, which is consistent with previous research on parents/caregivers living in remote or rural areas [24–27].

Before they will seek dental health care, individuals must be convinced that seeking dental care is more important than their other daily activities, including work [28]. The participants in this study needed to financially support their families; thus, they prioritized their work over dental care. This result was in line with the findings of previous studies that reported delayed dental health-seeking practices among caregivers because of other competing priorities in their lives [5, 17, 26, 29]. In our study, parents/guardians prioritized their work and students prioritized going to school, even if they had dental health problems. The lower perceived value of dental health, compared with general health, among the participants might be caused by dental health problems not being obvious and having relatively little impact on daily life activities. Furthermore, the low perceived value of dental health indicates that the participants had insufficient knowledge of the relationship between general health and oral health. A previous qualitative study among ethnic minority caregivers also identified their low prioritization of dental health as a reason for delays in seeking oral health care for their children [6].

In line with the findings of a previous study among parents and caregivers of preschool children [27], we found that fear of dental procedures was common among the student participants, who were afraid of pain during dental procedures such as tooth extraction. A previous qualitative study found that it was challenging for parents to take their children to the dental clinic if they feared dental treatments [26], and another study found that parents were unwilling to take their children to the dental clinic because of the children’s dental fear [17]. It may also be difficult to get these children to cooperate in visiting the dental clinic, leading to a lack of dental care utilization among children. Tooth extraction was a common treatment for the children in the present study because of their low dental care utilization. Therefore, the children often had personal experience with dental procedures that they found frightening or had heard of these kinds of experiences from their friends, which might lead them to fear of visiting the dental clinic, further discouraging their dental health care utilization.

This study explored folk knowledge, perceptions, and oral health-seeking behaviors among the Karen population living in the Thai border area. The information presented here is critical for designing effective oral health promotion programs among this population. However, a limitation of this study was that Mae Tan Sub-district, which was selected for this study, does not represent the Karen populations living in other areas of Thailand. In addition, the parents/guardians responsible for children’s oral hygiene and seeking health care in this study were mostly women; this study context thus represents predominantly the views of children and women but less men point of view.

## Conclusions

This qualitative study revealed the folk knowledge, perceptions, and beliefs regarding oral health among the Karen ethnic group living in the border area of Thailand. Building on the findings of this qualitative study, future research should focus on the improvement of knowledge regarding the consequences of dental diseases, appropriate treatment of dental diseases, and the importance of primary teeth in children, as well as testing the effectiveness of culturally appropriate oral health promotion programs among this community.

## Supplementary information

**Additional file 1**. Interview guide used for in-depth interview with adults and semi-structured interview with children.

**Additional file 2**. Questionnaire for obtaining demographic information of adult participants used in this study.

## Data Availability

The datasets generated and/or analyzed during the current study are not publicly available due to participant confidentiality restrictions but are available from the corresponding author on reasonable request.

## Abbreviation

RQDA: R package for qualitative data analysis
