# Supplementary material for: Perception and belief in oral health among Karen ethnic group living along Thai-Myanmar border, Thailand
Source: BMC Oral Health. 2020 Nov 11;20:322. doi: 10.1186/s12903-020-01318-w (PMC7659163; doi:10.1186/s12903-020-01318-w)
Supplement: Supplementary file 1 — Additional file 1. Interview guide used for in-depth interview with adults and semi-structured interview with children. [file 12903_2020_1318_MOESM1_ESM.docx]

# INTERVIEW GUIDE

**Perception of oral health**

- Opinion of healthy teeth and gum?
- Current situation of child’s oral health (Good, fair or poor? Why?)

# Oral health seeking behavior

- Child’s experience of dental pain (If yes, What did you do?, Why?)
- Experiences of visiting to the dental clinics because of child’s oral health problems (What?, Good or Bad?, Why?)
- Dental care decision maker for child in family (Who?)
- Reasons for visiting or not visiting to dental clinics (What?)
- Time to take child to dental clinics (Why?)

# Oral health knowledge

- Importance of primary and adult teeth (Why?, How?)
- Problems of teeth and gum (What?)
- Consequences of unhealthy gum and teeth (What?)
- Relationship between oral health and general health (If yes, How?)
- How do you think about your children first and second set of teeth?
- Prevention of tooth decay (How?)
- Prevention of gum disease (How?)
- Desire to eat particular food (What?, Why?)
- Role of diet in child’s oral health (Good food?, Bad food?)
- Effects of sugary foods and beverages on oral health
