# Supplementary material for: Perception and belief in oral health among Karen ethnic group living along Thai-Myanmar border, Thailand
Source: BMC Oral Health. 2020 Nov 11;20:322. doi: 10.1186/s12903-020-01318-w (PMC7659163; doi:10.1186/s12903-020-01318-w)
Supplement: Supplementary file 2 — Additional file 2. Questionnaire for obtaining demographic information of adult participants used in this study. [file 12903_2020_1318_MOESM2_ESM.docx]

**QUESTIONNAIRE FOR PARENTS/GUARDIANS/CHILDREN**

Thank you for your participation in the interview section. I would like to invite you to answer this survey before starting interview. It can help us to understand better about your background and your household characteristics.

Study ID______

1. What is your date of birth? Day ________ Month________ Year_________
2. Gender

( ) Male ( ) Female

1. Ethnic Group

( ) Karen ( ) Thai ( ) Myanmar

( ) Other (please specify) ________________

1. What is your religion?

( ) Buddhism ( ) Islam ( ) Christian ( ) None

1. Where were you born?

( ) Myanmar ( ) Thailand ( ) Others (Please specify) ________________

1. How long have you been living in Thailand?

( ) From birth ( ) From ______ years of age

1. What is your education level?

( ) Illiterate ( ) Primary school ( ) Secondary school

( ) Bachelor degree or higher

1. What do you do for a living now? (Check all that apply)

( ) Farming and gardening ( ) Casual laborer

( ) Hunting and collecting in forest ( ) Raising animals

( ) Government officer ( ) Fishing

( ) Cattle trading ( ) Trading ( ) Housewives

( ) Others (Please specify) ________________
